# A Comparative Metabolomics Study of Multiple Urological Diseases by Highly Sensitive Dansylation Isotope Labeling LC-MS

**DOI:** 10.3390/ijms262311353

**Published:** 2025-11-24

**Authors:** Wei-Hsuan Wang, Ya-Ju Hsieh, Chien-Lun Chen, Ying-Hsu Chang, Yi-Huai Tsai, Chih-Hsiang Chang, Liang Li, Wei-Ju Tu, Jau-Song Yu, Yi-Ting Chen

**Affiliations:** 1 College of Medicine, Chang Gung University, Taoyuan 333, Taiwan; will00613@yahoo.com.tw (W.-H.W.); changyinghsu@gmail.com (Y.-H.C.); 2 Medical Education Department, Chang Gung Memorial Hospital, Taoyuan 333, Taiwan; 3 Molecular Medicine Research Center, College of Medicine, Chang Gung University, Taoyuan 333, Taiwan; hsiehyaju@mail.cgu.edu.tw (Y.-J.H.); kopa92414@gmail.com (Y.-H.T.); yusong@mail.cgu.edu.tw (J.-S.Y.); 4 Department of Urology, Chang Gung Memorial Hospital, Taoyuan 333, Taiwan; 5 Department of Urology, New Taipei Municipal TuCheng Hospital, New Taipei 236, Taiwan; 6 Kidney Research Center, Department of Nephrology, Chang Gung Memorial Hospital, Taoyuan 333, Taiwan; franwisandsun@gmail.com; 7 Department of Chemistry, University of Alberta, Edmonton, AB T6G2G2, Canada; liang.li@ualberta.ca; 8 Graduate Institute of Biomedical Sciences, College of Medicine, Chang Gung University, Taoyuan 333, Taiwan; imtuweiju@gmail.com; 9 Department of Biomedical Sciences, College of Medicine, Chang Gung University, Taoyuan 333, Taiwan

**Keywords:** urinary metabolome, urinary tract diseases, metabolomics, isotope labeling LC-MS

## Abstract

Urine analysis is a straightforward, non-invasive testing method that, when integrated with metabolomics, shows great potential for detecting small-molecule metabolites as biomarkers of abnormal metabolic activity in the urinary tract, including drug interactions, toxicity, and diseases. However, integrated and comparative analyses of multiple urinary tract pathologies are currently limited. In this study, ^12^C2/^13^C2-chemical dansylation labeling was used to explore the urinary amine/phenol-metabolome profiles of eight urological conditions compared with normal profiles. We obtained ten samples for each condition (disease and normal) from a total of 90 participants, pooling them as representative samples, and constructed metabolite panels to differentiate various urological conditions. We discovered nine metabolites that were dysregulated between urine samples from patients with and without cancer. Another seven metabolites were differentially expressed between the benign prostatic hyperplasia group and the prostate cancer group. Among 1854 peak pairs of metabolites in an amine/phenol submetabolome analyzed by dansyl chloride derivatization coupled with LC–MS/MS, 1747 (94.2%) were detectable in urine specimens from all nine groups. Notably, 18 identified metabolites showed substantial stability across all urological conditions. Given the considerable variability in urine metabolite composition, these metabolites could potentially be used for normalization in urine metabolome analysis, addressing the need for stably expressed molecules as internal standards in the development of urinary biomarkers. Our findings provide the preliminary insights into the stability of urinary metabolomics and the metabolic perturbations associated with different urinary tract-related pathologies.

## 1. Introduction

Current clinical diagnostic methods for urinary system diseases include urine cytology, ultrasound, computed tomography (CT), magnetic resonance imaging (MRI), and endoscopy (e.g., cystoscopy, nephroscopy). While some are costly, invasive, and pose health risks [1]. urine cytology remains a non-invasive, specific, and cost-effective option, though its sensitivity for early-stage urological cancer is limited [2].

Metabolomics analyzes small-molecule compounds in biological fluids, capturing biochemical reactions and interactions with the environment, including pathogens. Given the metabolome’s dynamic complexity and the ease of fluid collection, metabolomics holds great promise for identifying reliable metabolic markers for early-stage urological disease diagnosis [3,4]. Unlike traditional urine analysis, modern metabolic analysis integrates all metabolic pathways for more comprehensive results. Urine metabolomics is categorized into metabolomic profiling [5,6], metabolite fingerprinting [7], and target metabolite analysis [8]. Profiling and fingerprinting provide detailed metabolome data, making them effective screening strategies [9]. Establishing comprehensive urinary profiles is essential for identifying metabolic pathways and distinguishing environmental, dietary, and pathogenic influences, aiding in early disease diagnosis and biomarker identification [10,11].

Liquid chromatography-mass spectrometry (LC-MS) is a widely used method in metabolomics but faces challenges due to the diverse chemical structures of metabolites [12]. High-performance chemical isotope labeling (CIL) enhances LC separation, MS sensitivity, and quantification accuracy by targeting specific functional groups [13]. For instance, ^12^C_2_/^13^C_2_ dansylation labeling improves profiling of amine- and phenol-containing submetabolomes. The hydrophobic and charged properties of the dansyl group enhance LC separation and ionization efficiency by electrospray ionization (ESI), improving MS intensity and signal-to-noise ratios [13]. Additionally, mixing ^12^C_2_-dansyl–labeled samples with ^13^C_2_-dansyl–labeled universal metabolite standards (UMS) ensures reliable metabolite identification. The resulting ^12^C_2_/^13^C_2_ peak pairs provide confident signals, and the peak area ratio offers more precise quantification than label-free methods. A recent study also suggests that LC-MS–based metabolomic profiling enables deep insights into biological systems and disease mechanisms. Ongoing advances in chromatography, MS technology, and data analytics have made it a reliable tool for large-scale biomarker discovery [4,14,15].

Metabolite analysis provides a precise tool for diagnosing urinary tract diseases. Previous studies have identified markers for conditions like urinary tract infection (UTI), benign prostatic hyperplasia (BPH), and prostate cancer (PCa) [16,17]. Urine metabolite analysis overcomes limitations of traditional tests, with NMR- and MS-based methods have been used to detect urinary tract cancer [18]. Efforts to standardize metabolomics procedures aim to enhance clinical applications [17], though comparative studies across different diseases remain limited.

Despite significant progress in urinary metabolomics, comparative profiling across multiple urological conditions using a unified analytical workflow remains limited. The present study provides the first comprehensive amine/phenol submetabolome comparison across eight common urological diseases using ^12^C_2_/^13^C_2_ isotopic dansyl labeling LC-MS. In addition, we introduce a universal metabolome standard (UMS)-based quantification platform that enables robust cross-cohort comparison [19]. Importantly, we identify highly stable urinary metabolites that may serve as endogenous normalization candidates—addressing a critical gap in urine metabolomics standardization. Furthermore, by contrasting malignant and benign conditions and directly comparing PCa and BPH, our study reveals shared and disease-specific metabolic perturbations that provide new insights into urological disease biology and biomarker discovery.

## 2. Results

### 2.1. Amine/Phenol-Metabolomics Profiling of Urine Samples from Nine Urinary Clinical Conditions Using CIL LC-MS

**Figure 1:**
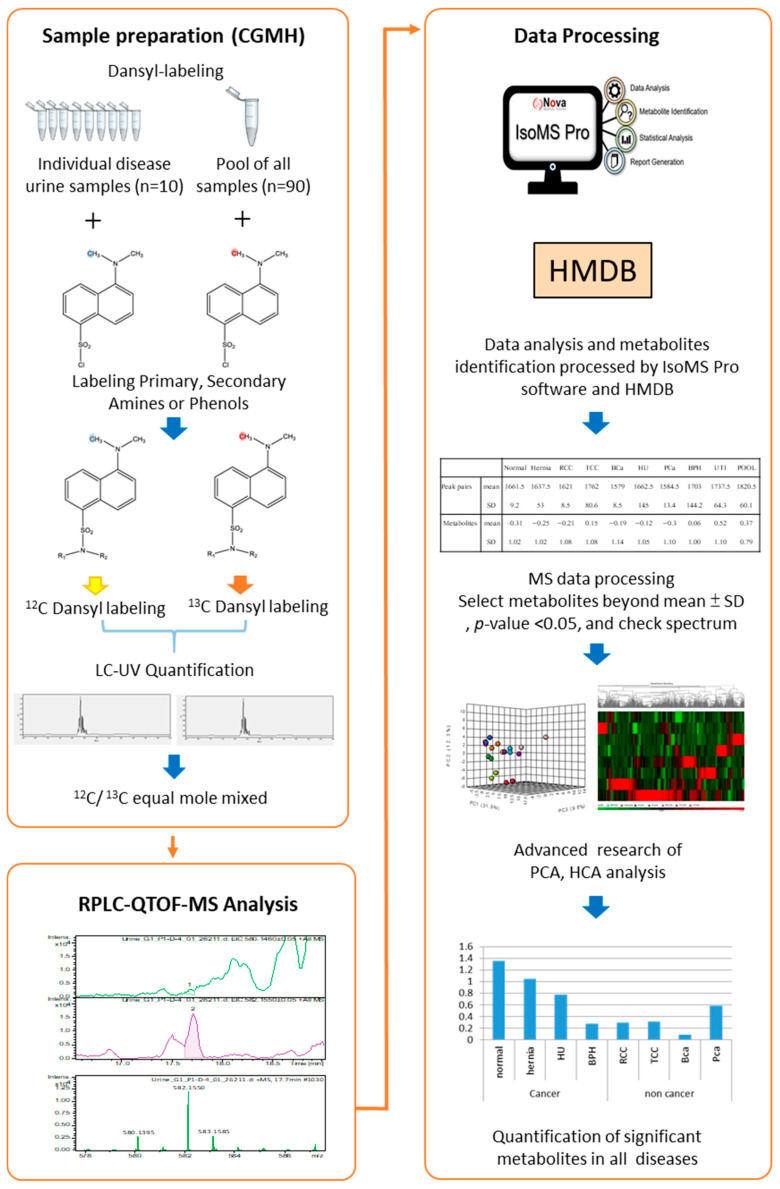
Workflow of dansyl labeling with a universal metabolite standard (UMS) platform for metabolomics profiling in urinary tract-related conditions (n = 10 individual urine samples per disease group).

Urine metabolomes were compared across major urological conditions, including BCa, PCa, RCC, TCC, hematuria, UTI, BPH, and hernia, alongside normal controls. Each clinical group’s 10 pooled samples were labeled with ^12^C_2_-DNSC for analysis. Given the variations in metabolite content and concentration among individuals, a quantitative normalization analysis was performed and a universal metabolite standard (UMS) labeled with ^13^C_2_-DNSC served as an internal standard. LC-UV quantified DNSC-labeled metabolites, ensuring equal input for LC-MS analysis. Fixed molar amounts of ^12^C_2_-labeled metabolites and equal ^13^C_2_-labeled UMS were injected into the LC-MS system, with the ratio of ^12^C_2_/^13^C_2_-labeled metabolites providing relative quantitative results for numerous metabolites in urine samples from the indicated clinical conditions. We have previously published several quantitative metabolomics studies [14,19,20] using the UMS strategy, in which the pooled UMS sample contains metabolites originating from all clinical samples. Because the same amount of UMS metabolite is added to each individual sample, it serves as an internal standard to enable accurate relative quantification. This approach has been demonstrated to provide high analytical consistency and comparability across samples. IsoMS Pro identified differentially expressed metabolites, distinguishing cancerous from non-cancerous urinary conditions. Figure 1 illustrates the dansyl-labeling metabolomics workflow.

**Figure 2:**
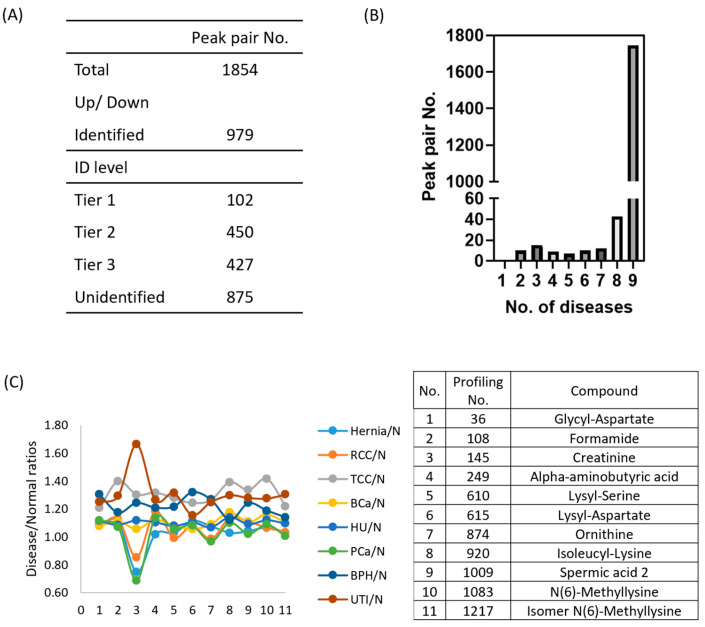
Performance of metabolomics profiling across nine urinary conditions, based on pooled samples from individual urine samples (n = 10) per disease group. (**A**) Numbers of metabolite peak pairs detected and identified using IsoMS Pro, categorized by identification Tiers (Tier 1, 2, 3) and unidentified metabolites. (**B**) Distribution of detected metabolites across different urine specimens. A total of 1747 metabolites were detected in all nine clinical conditions, with 43 metabolites unique to eight urine specimens. (**C**) Concentration levels of 11 stably expressed metabolites (with identities) in pathological urine compared to normal urine, with corresponding profiling numbers and compound names listed in the right panel.

^12^C_2_/^13^C_2_ labeling enhances the detection and quantification of amine and phenol metabolites in mass spectra. By using ^13^C_2_-labeled pooled samples as a UMS, ^12^C_2_-labeled metabolites in urine from different clinical conditions were relatively quantified through ^12^C_2_/^13^C_2_ ratio [21]. Our analysis using IsoMS Pro software detected 1854 peak pairs of metabolites in urine samples representing nine clinical conditions (Table S1), with each condition exhibiting 1500–1800 peak pairs (Table S2). On average, 1677 ± 59 amine/phenol-containing metabolites were identified. Among these, 979 amine/phenol-containing metabolite peak pairs were identified, with 102 classified at Tier 1, 450 at Tier 2, and 427 at Tier 3. Tier 1 metabolites were confidently identified by matching accurate mass and retention time to labeled standards. Tier 2 metabolites were identified with high confidence using accurate mass and predicted retention time from a reference library. Tier 3 metabolites were putatively identified based on accurate mass matching against the MyCompoundID (MCID) zero-reaction metabolome (~8000 endogenous human metabolites from HMDB). However, 875 peak pairs remain unidentified (Figure 2A). We also aimed to discover differentially expressed metabolites in various conditions (compared to pooled urine samples) by calculating log_2_-transformed ratios of means ± SD (Table S2). Notably, UTI samples had the highest log_2_ ratio (0.52 ± 1.10), suggesting increased metabolite levels due to bacterial infection, while normal urine had the lowest (−0.31 ± 1.02). Further details are available in Table S2.

### 2.2. Evaluation of Potential ‘Housekeeping Metabolites’ in Urine Specimens from Nine Urinary Clinical Conditions

Despite the potential of urine metabolomics screening, metabolite variability presents challenges for research consistency [22]. Normalization using ‘housekeeping’ metabolites, which remain stable despite external influences, can help overcome this issue [23]. Common approaches include osmolality-based normalization and pre-acquisition calibration using parameters like creatinine [24]. However, creatinine normalization is affected by factors such as age, gender, kidney function, and body mass [25], while osmolality-based normalization may be influenced by medications or organ diseases [26], often requiring larger sample volumes [27]. Given the effects of urinary tract diseases on osmolality and creatinine, identifying stable housekeeping molecules with minimal variation is essential for reliable normalization and broader clinical applications.

We identified metabolite molecules with minimal concentration variability across urine samples from nine urinary clinical groups using a consistent analytical platform and UMS for accurate quantification. Of the 1854 metabolite peak pairs detected in the amine/phenol submetabolome via dansylated LC-MS, 1747 (94.2%) were present in all clinical groups (Figure 2B). Among these, 18 metabolites remained stably expressed across pathological conditions compared to normal urine, based on a log_2_-transformed mean ± 0.2SD threshold (Table S3). These metabolites have potential as endogenous calibration compounds for improved normalization methods. Among them, 11 identified metabolites and creatinine were analyzed for their concentration levels in urine samples (Figure 2C). Notably, creatinine remained stable in RCC, BCa, and HU (0.8–1.2 range) but exhibited lower levels in hernia and PCa and higher levels in TCC, BPH, and UTI. The other 11 metabolites appeared to be more stable than creatinine in the eight urinary clinical samples. Detailed information on these 18 metabolites is provided in Table S3.

### 2.3. Unsupervised Statistical Analysis of the Urine Submetabolome from the Nine Urological Groups

**Figure 3:**
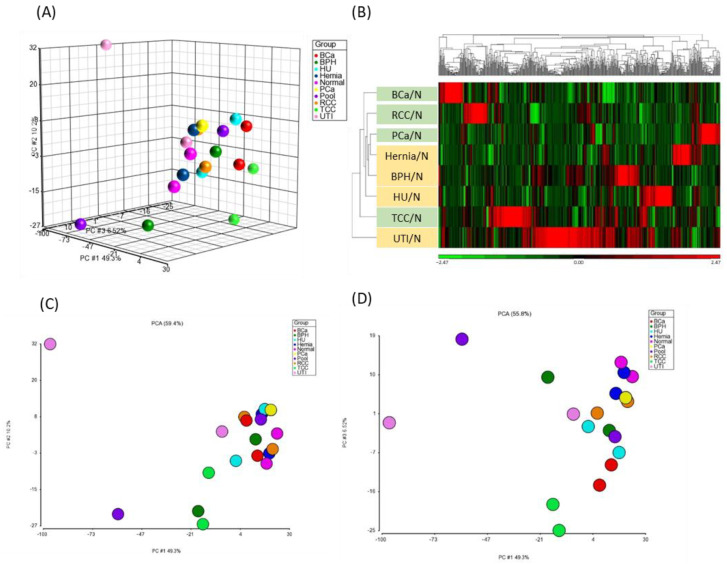
PCA and HCA analyses of metabolomics data. A total of 35,226 concentration ratios of all metabolite peak pairs from 100 urine samples were analyzed by PCA and HCA. (**A**) PCA results, with samples segregated based on urinary conditions: BCa (red), BPH (green), hernia (dark blue), HU (light blue), normal (pink), PCa (yellow), Pool (purple), RCC (orange), TCC (light green), and UTI (light pink). (**B**) HCA heat map of 520 metabolites with differentially regulated disease-to-normal ratios (>mean ± 2SD). Each column represents a metabolite, and each row represents a urinary condition. The color scale depicts log2-transformed values, indicating relatively high (red) and low (green) metabolite levels. (**C**,**D**) presented the 2D score plots of PC1 vs. PC2 and PC1 vs. PC3.

Principal Component Analysis (PCA) was employed to examine variations in urinary metabolomes among the nine clinical conditions (eight urological pathologies and one normal control group). Concentration ratios of light-labeled to heavy-labeled metabolites of all peak pairs were utilized for PCA (Figure 3A) using MetaboAnalyst 6.0. PCA did not show significant separation between normal and malignancy groups. While PCA did not clearly distinguish normal from malignant cases, PC1, PC2, and PC3 showed improved discriminatory power, particularly separating UTI from other conditions. Common biological processes such as inflammatory responses have been implicated in both benign (hernia, UTI) and malignant (cancers) diseases [28]. Notably, metabolites in the tryptophan metabolic pathway, including serotonin and kynurenine, play key roles in inflammation and cancer [29,30]. Our analysis detected tryptophan, tryptophanamide, kynurenine, 3-hydroxy-kynurenine O-glucuronide, and N2-acetyl-L-kynurenine, all upregulated in hernia, UTI, and cancer samples. These metabolites, integral to essential biological pathways, may contribute to the complexity of disease discrimination solely through PCA.

Hierarchical clustering analysis (HCA) was performed to identify differentially expressed metabolites (i.e., >means ± 2SD) in each disease compared to normal urine samples. A total of 313 metabolites met the criteria (highlighted in yellow in Table S4). Among them, 5 metabolites showed increased levels and 19 showed decreased levels beyond mean ± SD across all urine samples of the eight diseases. Additionally, specific alterations were observed in metabolites unique to each disease, as illustrated in Table S4. Consistent with PCA findings, HCA revealed distinct clustering patterns, particularly emphasizing the unique metabolite composition of UTI urine samples, which displayed greater dissimilarity from other groups (Figure 3B). This suggests that UTI, characterized by infection and inflammation in the urinary tract, induces distinct immune responses that modulate urinary metabolite profiles. Similar clustering patterns have been reported in previous urine metabolomics studies, where UTI samples formed distinct clusters due to bacterial metabolic activity, particularly *E. coli*–derived metabolites such as PABA and aromatic amines. Moreover, malignant urine samples (BCa, PCa and RCC) were notably segregated from non-cancerous samples by HCA (Figure 3B). It is consistent with previous clinical metabolomics studies, which have demonstrated reproducible cancer-specific urinary signatures [31,32,33,34,35,36,37]. Prostate, bladder, and renal cancers show characteristic alterations in pathways such as choline metabolism, tryptophan–kynurenine metabolism, the TCA cycle, and lipid metabolism, enabling clear separation from non-cancer groups. These findings support the biological and diagnostic relevance of the cancer-related clusters identified in our study which further underscoring potential biomarkers for cancer diagnosis [38,39].

### 2.4. Comparison of Metabolome Composition Between Urine Specimens from Individuals with and Without Cancer

CIL has been employed to investigate metabolic changes in cancer cells and to identify specific target metabolites in biofluids [40]. However, no prior study has examined metabolomic alterations across multiple cancers and their interrelationships. Therefore, we aimed to identify common metabolic pathway alterations in urine specimens from patients with various cancers by comparing their metabolomic profiles with those of benign conditions.

**Figure 4:**
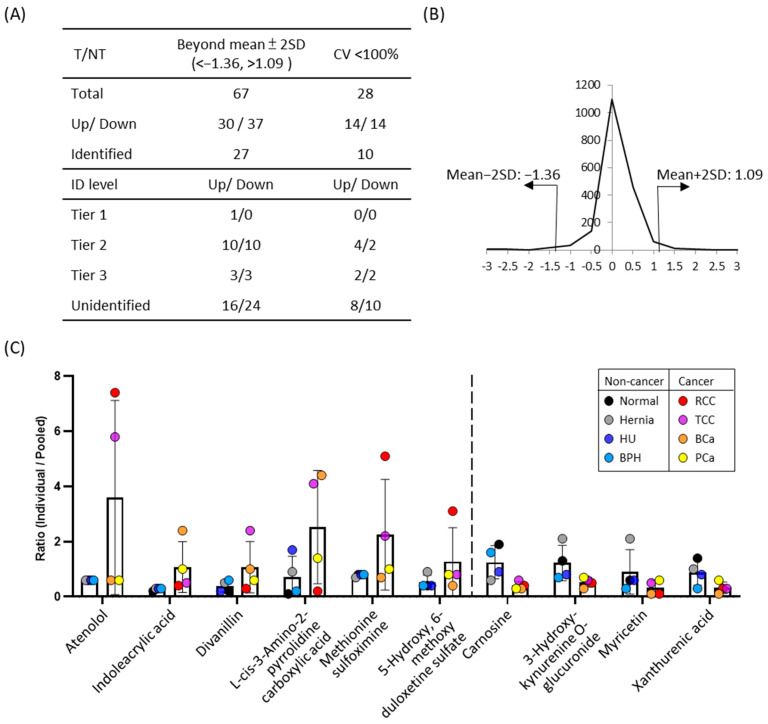
The results of comparative amine/phenol-metabolome between cancer and non-cancer urine samples. (**A**) Metabolite ratios of the cancer group were averaged and compared to the non-cancer group. Fold-changes between two groups were calculated, and metabolites showing differences greater than the mean ± 2SD were considered significantly altered. Data were processed using IsoMS Pro, with metabolite identifications categorized into Tier 1, Tier 2, and Tier3 levels. The coefficient of variation (CV) was assessed for both cancer and non-cancer groups, and metabolites with CV < 100% are presented. (**B**) Distribution of fold changes (log_2_ transformed) between cancer and non-cancer groups. Thirty metabolites were significantly increased (<mean ± 2SD), 14 with identities. Thirty-seven metabolites were significantly decreased (>mean ± 2SD), 13 with identities. Ten metabolites with identities and CV < 100% were illustrated in (**C**). Ratios of metabolites relative to pooled samples in both cancer and non-cancer groups. The six metabolites on the left were elevated in the cancer group compared to the non-cancer group, whereas the four metabolites on the right were reduced in the cancer group.

Given the distinct metabolomics profile observed in UTI urine, likely influenced by substantial bacterial metabolite production (Figure 3B), and the distribution of metabolite concentrations was broader than that observed in other disease groups (Figure S1). In addition, several immune-related metabolites were elevated in UTI urine, such as N2-Acetyl-L-Kynurenine (No. 486, FC of UTI/N = 1.75) and formyl-5-hydroxykynurenamine (No. 1339, FC of UTI/N: 1.34) (Table S1). Polyamines have been reported to accumulated in *E. coli*-induced UTIs [41], and consistent with this, dehydrospermidine was also increased in the present study (No. 714, FC of UTI/N: 1.5). To minimize potential confounding effects, UTI samples were excluded from subsequent analyses. We then categorized urine samples into two groups: the tumor (T) group, comprising RCC, TCC, BCa, and PCa patients, and the non-tumor (NT) group, including normal individuals and patients with hernia, BPH, and HU. To identify differentially expressed metabolites between cancerous and non-cancerous conditions, we calculated the T/NT fold change in all metabolite peak pairs. Metabolites with fold changes exceeding the mean ± 2SD were classified as significant differential metabolites (Figure 4A,B). Assuming a normal distribution of log2-transformed concentrations, values outside the mean ± 2 SD range are considered statistically significant at approximately *p* < 0.05 (two-tailed). Of the 67 significantly altered metabolites (30 upregulated, 37 downregulated), 27 were putatively identified using IsoMS Pro processing based on Tier 1–3 criteria [42]. To ensure reliability across cancer and non-cancer groups, we further refined our selection, retaining only metabolites with identifications and a coefficient of variation (CV) < 100%. This resulted in 10 metabolites (6 upregulated, 4 downregulated) that met these criteria (Table S5). These metabolites were identified at Tier 2 or 3 levels, requiring additional validation before their potential use as clinical biomarkers.

Among these 10 candidate metabolites, the urinary concentrations of atenolol, indoleacrylic acid, divanillin, L-cis-3-amino-2-pyrrolidinecarboxylic acid, methionine sulfoximine, and 5-hydroxy-6-methoxy duloxetine sulfate were increased in cancer groups compared to non-cancer groups. Conversely, the four metabolites whose urinary concentrations decreased in cancer groups were carnosine, 3-hydroxy-kynurenine o-glucuronide, myricetin, and xanthurenic acid (Figure 4C). In particular, atenolol was significantly elevated in RCC and TCC urine samples. Methionine sulfoximine also showed significant increased ~6.5-fold in RCC and ~2.7-fold in TCC compared to normal urine (Figure 4C). Divanillin concentrations increased 3.3 times in cancer urine, with a notable ~15-fold rise in TCC patients. (Figure 4C and Table S5). On the other hand, carnosine levels were lower in cancer urine specimens, with a concentration ratio of 0.38-fold compared to the normal group (Figure 4C). Similarly, 3-hydroxy-kynurenine O-glucuronide levels in the cancer group were ~0.4-fold those in the normal group. Xanthurenic acid also decreased to 0.3-fold of NT group level. The trends of increased metabolites appeared to be disease-specific, while decreased metabolites showed similar patterns across multiple cancer types. As pooled samples were used in this study, further validation using individual specimens is needed to confirm these findings and assess their diagnostic potential.

### 2.5. Comparison of the Urinary Metabolome Between BPH and PCa Patients

**Figure 5:**
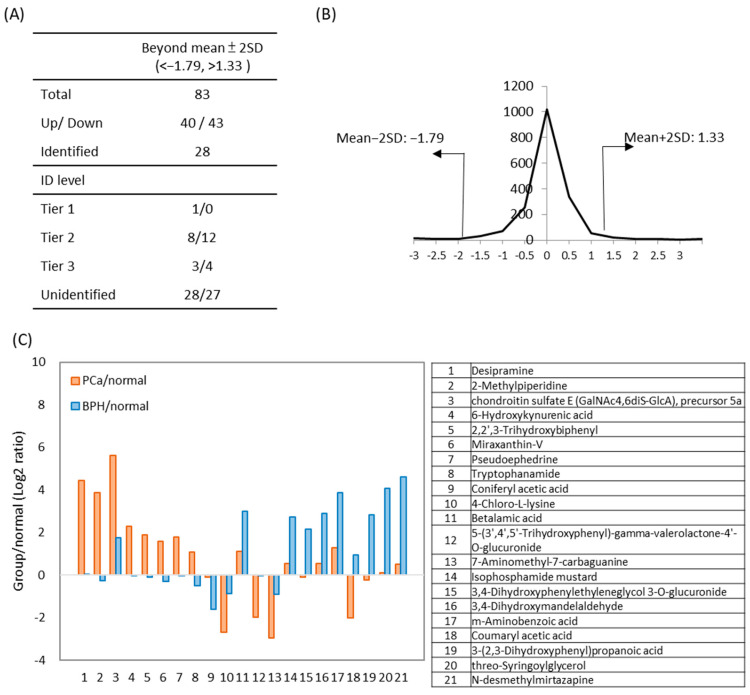
The results of comparative amine/phenol-metabolome between PCa and BPH urines. Metabolite ratios in the PCa group were compared to those in the BPH group, with significant differential concentration defined as fold-change > mean ± 2SD). (**A**) The number of metabolites identified during data processing, along with their identification levels. (**B**) Distribution of fold changes (log_2_ transformed) between PCa and BPH. (**C**) Twenty-one differential metabolites between PCa and BPH urine samples are presented. The ratios represent the levels of metabolites in PCa (orange) or BPH (blue) compared to normal urine samples. Compound names are listed below.

To compare the differences in metabolites between BPH and PCa patients, a comparative metabolomics analysis of urine samples was performed. Metabolites with significantly altered PCa/BPH concentration ratios (>mean ± 2SD) were selected for further investigation. (Figure 5). The distribution of fold-changes between PCa and BPH is shown in Figure 5B. Among the 83 differential metabolites, 28 were identified through database matching including 12 increased and 16 decreased metabolites (Table S6). The log_2_ PCa/BPH ratios of 21 additional metabolites identified with high confidence (Tier 1 and 2) are showed in Figure 5C. A manual assessment of the LC/MS chromatogram and spectra of PCa and BPH urine samples (Figure S2) suggests that these metabolites have the potential to differentiate PCa from BPH patients, pending further validation with a larger number of clinical specimens.

Cancer cells often exhibit an increased amount of kynurenine and its derivatives, tryptophan [30]. In PCa urine, 6-hydroxykynurenic acid was 4.9-fold higher than in BPH (Figure S2B). Additionally, desipramine was uniquely detected in PCa urine specimens, suggesting potential specificity. As Tier 2 identifications, these findings require further validation. Moreover, many unidentified peak pairs showed significant alterations between PCa and BPH urines. Identifying these unknown metabolites will be a key focus of future studies.

## 3. Discussion

In this study, we demonstrated the application of a CIL method, specifically an isotopic dansyl-labeling metabolomic platform, to profile the amine- and phenol-based submetabolome in urine samples from healthy individuals and patients with one of eight urinary tract conditions. To address inter-sample variability, we pooled 10 specimens per group and employed LC-UV and CIL LC-MS normalization, enabling the stable detection of 1600–1800 peak pairs in each run (Table S2). Additionally, we utilized a ^13^C_2_-DNSC-labeled universal pooled standard as a control to allow for the relative quantification of detected metabolites, overcoming hydrophilicity-related challenges and enhancing metabolite detection. Given the absence of conclusive normalization methods for biomolecule quantification in clinical urine specimens, there is an urgent need to discover additional molecules that are widely and stably expressed in urine. Leveraging the precise quantification provided by our platform, we identified 18 consistently expressed metabolites (11 with known identities) as potential normalization biomarkers. These compounds represent promising candidates for endogenous normalization, offering a more physiologically grounded alternative to traditional normalization methods such as creatinine or osmolality, both of which can be confounded by renal function, hydration status, and inflammation. This also highlights the robustness of CIL-based approaches for identifying potential metabolites that may be useful for biological parameter-based normalization.

Moreover, in Table S1, which lists a total of 1854 identified metabolites, we have included the corresponding DrugBank identifiers (https://go.drugbank.com/) for compounds matched in the database, to help readers assess the potential presence of drug-derived metabolites. In addition, we performed a statistical analysis of age among groups to evaluate potential age-related confounding effects, with the results presented in Figure S1. As shown in Figure S1, the distribution of urinary metabolite levels in each disease was compared with that of the normal group. Most metabolites exhibited fold changes close to 1 in the hernia versus normal comparison, indicating that the urinary metabolomic profile of the hernia group was indeed the most similar to that of the normal group.

To deepen the biological interpretation of our findings, we examined the functional implications of the significantly altered metabolites. Several metabolites associated with the kynurenine–tryptophan pathway, including 3-hydroxy-kynurenine O-glucuronide and xanthurenic acid, were consistently dysregulated across urinary cancers. HCA demonstrated distinct urinary metabolomics differences between cancer and non-cancer patients (Figure 3). Among these variations, we identified six increased metabolites and four decreased metabolites (Figure 4). Notably, the kynurenine pathway, which supports tumor survival and metastasis [43], was significantly altered. Cancer urine exhibited a 0.4-fold reduction in 3-hydroxy-kynurenine O-glucuronide and 4.9-times higher in PCa compared to BPH urine. Previous research suggests that the knockout of glucuronosyltransferase genes associated with PCa progression disrupts kynurenine metabolism, which may contribute to these metabolic shifts [44]. Since this pathway is known to modulate immune suppression, redox imbalance, and tumor progression [45], our findings suggest that abnormal concentrations of 3-hydroxy-kynurenine O-glucuronide in urine may reflect metabolic reprogramming in tumor cells, indicating urinary tract malignancy. Moreover, kynurenine-related metabolites could serve as potential biomarkers for distinguishing PCa from BPH.

Taking advantage of the diversity in our samples, we examined metabolomics differences between BPH and PCa patients (Figure 5C). BPH urine exhibited significantly higher levels of 3,4-dihydroxyphenylglycol (7.0-fold) and m-aminobenzoic acid (5.9-fold) compared to PCa. 3,4-Dihydroxyphenylglycol is a metabolic derivative found in human blood and *Escherichia coli* (strain K12, MG1655) [46], while m-Aminobenzoic acid, also known as para-aminobenzoic acid (PABA), is involved in bacterial folic acid synthesis and has been implicated in BPH risk [47]. Current evidence indicates that PABA plays a role in *E. coli* metabolism and is considered a possible risk factor for BPH. A previous study that screened 961 random radiolabeled molecules for essential metabolic pathways in bacteria found that PABA uptake specificity in *Staphylococcus aureus* and *E. coli*, suggesting metabolism-derived specificity [48]. Given the known link between bacterial-induced inflammation and BPH, PABA might have potential diagnostic applications in the early stage of BPH [49]. These findings highlight the biological connection between microbial activity and urinary metabolite signatures and the possible role of *E. coli* in BPH, supporting the use of LC-MS for detecting *E. coli*-related metabolites in urine for differential diagnosis.

3,4-Dihydroxymandelaldehyde, a neurotoxic phenyl acetaldehyde [50], was significantly elevated in BPH urine (7.62-fold higher than in PCa). Phenyl acetaldehydes are oxidized by NADP+-dependent aldehyde dehydrogenases (ALDHs), and impaired ALDH function may contribute to its accumulation. Given that ALDH1A1, ALDH7A1, and ALDH3A1 are upregulated in PCa and linked to tumorigenesis [51]. We speculated that the up-regulation of ALDH in PCa patients might lead to diminished levels of 3,4-dihydroxymandelaldehyde. Conversely, 2-methylpiperidine was markedly elevated in PCa urine (17.8-fold vs. BPH). A related compound, 1-methylpiperidine, exhibits high affinity for the σ1 receptor, whose overexpression in tumor cells is linked to poor prognosis and metastasis [52,53]. Elevated levels of methylpiperidine in urine have also been reported in PCa patients [52].

## 4. Materials and Methods

### 4.1. Selection and Pretreatment of Urine Samples

Urine specimens were collected from 90 patients, including 10 individuals for each of eight urinary tract-related conditions—UTI, BPH, PCa, bladder cancer (BCa), hernia, hematuria (HU), renal cell carcinoma (RCC), transitional cell cancer (TCC) along with one normal control group. Samples were obtained from Linkou Chang Gung Memorial Hospital, Taoyuan, Taiwan. The collection process was approved by the Institutional Review Board of the Chang Gung Medical Foundation (IRB Nos. 201601720B0 and 201601197B0), which is organized and operates in accordance with Good Clinical Practice guidelines and all applicable laws and regulations. Written informed consent was obtained from all participants and/or their legal guardians prior to sample collection. The inclusion criteria for the normal control group were as follows: (1) no history of urological diseases or systemic conditions that could affect urine composition, and (2) normal urine analysis results. All participants were Taiwanese and were required to undergo a 12 h overnight fast before providing their first morning urine sample for analysis. The first void of morning urine from patients was processed as described in our previous study. Samples were processed, centrifuged, filtered, and stored at −80 °C [19]. Age-matched specimens stored for under a year were used for metabolomics experiments. Demographic information of patients is shown in Table 1 and Table S7. Representative urine samples for each clinical group were prepared by pooling 10 μL of urine from each of the 10 patients in that group (10 samples/group × 9 groups = 90 samples). To facilitate metabolome comparisons among multiple sample sets in subsequent isotopic dansyl-labeling metabolomic experiments, a Universal Metabolome Standard (UMS) was prepared by mixing 10 μL of each separate pool (total volume, 100 μL), as described previously [19,21].

### 4.2. Reagents

Compounds for dansylation including dansyl chloride (DNSC), sodium bicarbonate (NaHCO_3_), sodium carbonate (Na_2_CO_3_), sodium hydroxide (NaOH), formic acid (FA) and the amino acid standards (AAS 18) were purchased from Sigma–Aldrich (St. Louis, MO, USA). Acetonitrile with 0.1% formic acid, water with 0.1% formic acid, MS grade water, and acetonitrile were also obtained from Sigma–Aldrich for the LC-MS buffer system. ^13^C_2_-dansyl chloride (^13^C_2_-DNSC) was sourced from TMIC (The Metabolomics Innovation center; Edmonton, AB, Canada).

### 4.3. Dansylation of Metabolite Extracts

The comparative metabolomics procedure using ^12^C_2_/^13^C_2_-dansyl labeling follows our previous study [21]. For each clinical condition, a 12.5 μL sample was mixed with 37.5 μL H_2_O, followed by the addition of 25 μL of sodium carbonate/bicarbonate buffer (0.25 M, pH 9.5), 25 μL of acetonitrile, and 50 μL of freshly prepared ^12^C_2_-dansyl chloride (DNSC, light isotope, 18 mg/mL in acetonitrile). The mixture was incubated at 40 °C for 45 min, quenched with 10 μL of 250 mM sodium hydroxide, and pH-adjusted with 50 μL of 425 mM formic acid. Samples for the nine clinical conditions were labeled with ^13^C_2_-DNSC [19].

### 4.4. Quantification of Amine/Phenol Submetabolome in Individual Urine Specimens

The concentration of dansylated metabolite extracts from each sample was measured using an LC-UV method reported in a previous study. A standard curve for quantification of dansylated metabolites was prepared by labeling a serially diluted (0.05 mM, 0.10 mM, 0.20 mM, 0.30 mM, 0.60 mM, 1.20 mM, 1.80 mM, 2.00 mM, 2.60 mM, and 5.20 mM) amino acid standard mixture (AAS18) with ^12^C_2_-DNSC (18 mg/mL). The UPLC system (Milford, MA, USA) employed a reversed-phase ACQUITY BEH C18 column (2.1 mm × 50 mm, 1.7 μm particle size, 130 Å pore size; Waters, Milford, MA, USA). Solvent A was 0.1% FA in H_2_O, and solvent B was 0.1% FA in acetonitrile. The flow rate was set to 250 μL/min with a 6 min gradient (0 min, 10% solvent B; 1.00 min, 10% solvent B; 1.01 min, 95% solvent B; 2.50 min, 95% solvent B; 3.00 min, 5% solvent B; 6.00 min, 10% solvent B). UV absorbance at 338 nm was recorded. Following LC-UV analysis, area and concentration data were used to construct a standard curve with an R-squared value > 0.99, enabling quantification of DNSC-labeled urine metabolite samples. Equal amounts of amine- and phenol- metabolites from each individual urine sample were then injected into LC-MS for metabolomic comparison. Subsequently, accurately quantified ^12^C_2_- and ^13^C_2_-DNSC labeled amine/phenol metabolites were injected into LC-MS for sample analysis.

### 4.5. LC-MS Analysis and Data Processing

Each sample was analyzed by injecting a 90 nmol metabolite mixture containing equal amounts of ^12^C_2_- and ^13^C_2_-DNSC labeled amine/phenol metabolites into the LC-MS system. A QC sample pooled from 90 individual urine samples was analyzed together with the nine group samples. LC-MS analyses were performed using an Agilent 1290 HPLC system (Palo Alto, CA, USA) coupled with a Bruker HD Impact II quadrupole time-of-flight (QTOF) mass spectrometer (Billerica, MA, USA). The flow rate was set at 60 μL/min with a 34 min gradient: t = 0 min, 10% B; t = 2 min, 10% B; t = 3 min, 20% B; t = 13 min, 38% B; t = 25 min, 71% B; t = 28 min, 99% B; and t = 32.5 min, 99% B. The Q-TOF MS parameters included positive ion polarity, a mass range of m/z 120–1150, spectral rate of 1 Hz, and resolution of approximately 30,000 at m/z 1221. ^12^C_2_/^13^C_2_-DNSC labeled metabolites were detected as peak pairs with mass intensity greater than 2000, and their light/heavy peak ratios were aligned and quantified using IsoMS Pro software (V.1.4.0, Nova Medical Testing, Edmonton, AB, Canada) [54,55]. Metabolites were identified through a three-tier system [56]: Tier 1 matched metabolites to a labeled standard library based on accurate mass and retention time; Tier 2 used a Linked Library with predicted retention times; and Tier 3 identified metabolites solely by accurate mass using the human metabolites database (HMDB) database with a 50 ppm mass tolerance and retention time window of 1 min.

### 4.6. Statistical Analysis

Metabolite peak pair ratios processed with IsoMS Pro were log2-transformed, and fold changes were calculated. *t*-tests and unsupervised statistical analyses were then conducted. Principal component analysis (PCA) and hierarchical clustering were performed using MetaboAnalyst 6.0 and Partek Genomics Suite (v6.6; Partek Inc., St. Louis, MO, USA), respectively.

## 5. Conclusions

This study utilized ^12^C_2_/^13^C_2_ differential dansyl chloride labeling to profile urinary metabolomics across eight common urinary tract conditions. We identified 18 stable metabolites and suggest their potential use for normalization in the quantification of urinary biomolecules. Six metabolites were elevated and four were reduced in cancer patients compared to non-cancer groups. A comparative analysis of amine/phenol metabolomes between BPH and PCa urine revealed 12 metabolites elevated in BPH and nine in PCa. Our findings underscore the role of kynurenine-derived metabolites in malignancy and suggest a stronger link between E. coli metabolism and BPH, highlighting potential biomarkers for BPH-related inflammation. We proposed several candidate metabolite biomarkers for diagnosing urinary tract conditions and identified stable metabolites for specimen normalization. This study has a limitation due to its limited sample size, which only enables us to create a preliminary yet accurate quantitative map of the deep metabolome across various urological diseases. In the future, it will be essential to utilize validation cohorts to achieve further absolute quantification of metabolites that demonstrate significant stability or disease specificity, providing a solid foundation for clinical application.

## Figures and Tables

**Table 1:** Baseline characteristics of the study population. This table presents the characteristics of clinical urine samples from the nine clinical conditions included in the metabolomics analysis.

Sample Group	Number of Samples	Age Range	Mean ± S.D.	Males	Females
Normal	10	20–21	20.5 ± 0.5	10	0
BCa	10	36–77	60.1 ± 13.4	10	0
BPH	10	50–79	63.7 ± 8.9	10	0
Hernia	10	48–79	66.3 ± 9.0	10	0
HU	10	23–74	49.0 ± 13.7	10	0
PCa	10	56–76	67.6 ± 6.6	10	0
RCC	10	39–77	61.9 ± 12.3	10	0
TCC	10	47–83	68.6 ± 9.6	7	3
UTI	10	49–66	58.4 ± 6.8	7	3

## Data Availability

The result files of LC MS/MS data for the urinary amine/phenol-metabolome profiles of eight urological conditions have been deposited in MassIVE FTP server (massive.ucsd.edu). Official URL for this dataset is: https://massive.ucsd.edu/ProteoSAFe/dataset.jsp?accession=MSV000097615 (accessed on 13 April 2025). To view the dataset’s files, log in to the MassIVE FTP server with this URL: ftp://MSV000097615@massive.ucsd.edu (accessed on 13 April 2025). (the Username for FTP access: MSV000097615 and password: CGUMMRC).

## Supplementary Materials

The supporting information can be downloaded at: https://www.mdpi.com/article/10.3390/ijms262311353/s1.
