# Supplementary material for: A Comparative Metabolomics Study of Multiple Urological Diseases by Highly Sensitive Dansylation Isotope Labeling LC-MS
Source: Int J Mol Sci. 2025 Nov 24;26(23):11353. doi: 10.3390/ijms262311353 (PMC12691989; doi:10.3390/ijms262311353)
Supplement: Supplementary file 1 [file ijms-26-11353-s001.zip › Figure S1 and S2_20251103.pptx]

## Slide 1
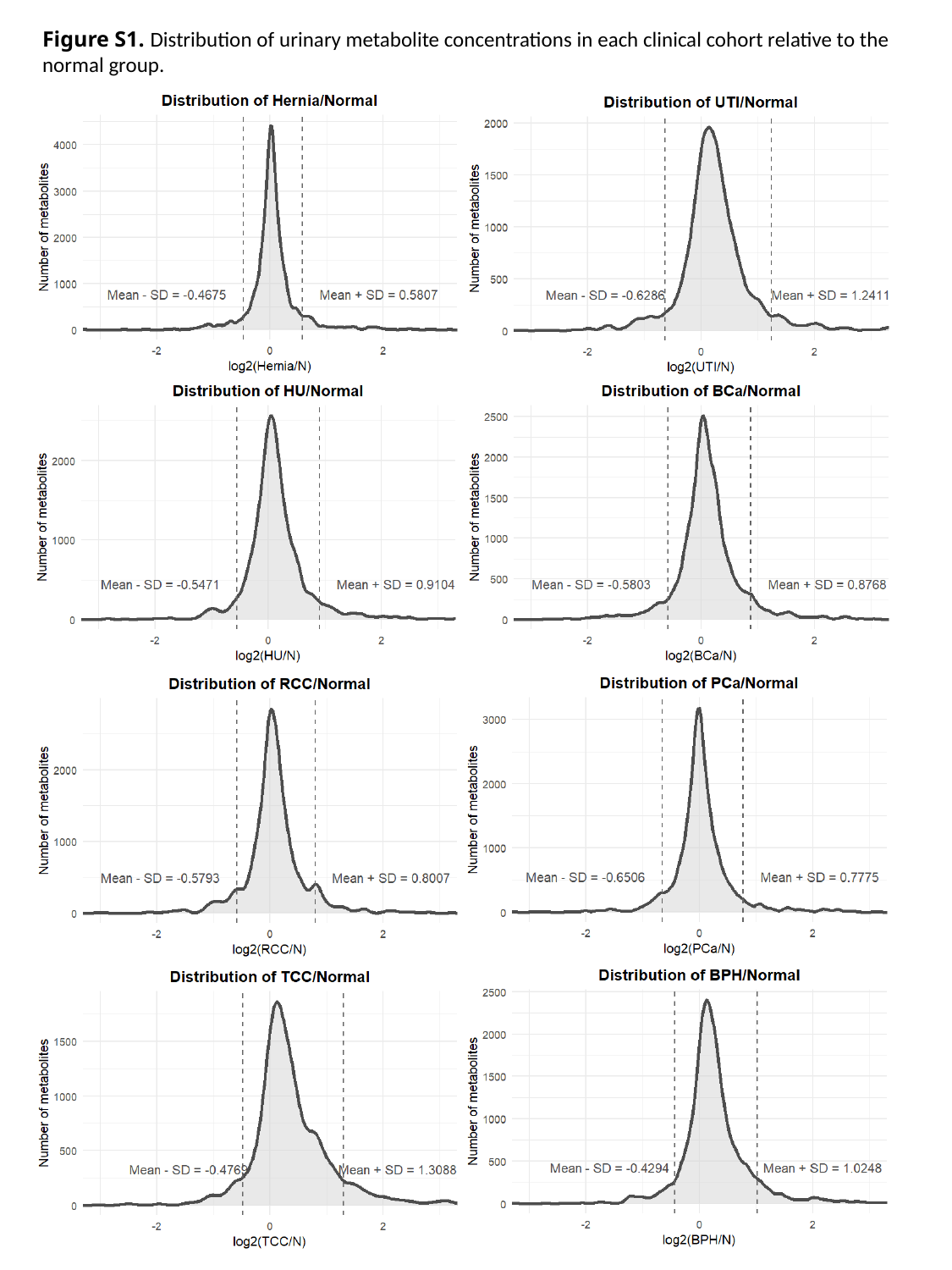

Figure S1. Distribution of urinary metabolite concentrations in each clinical cohort relative to the normal group.

## Slide 2
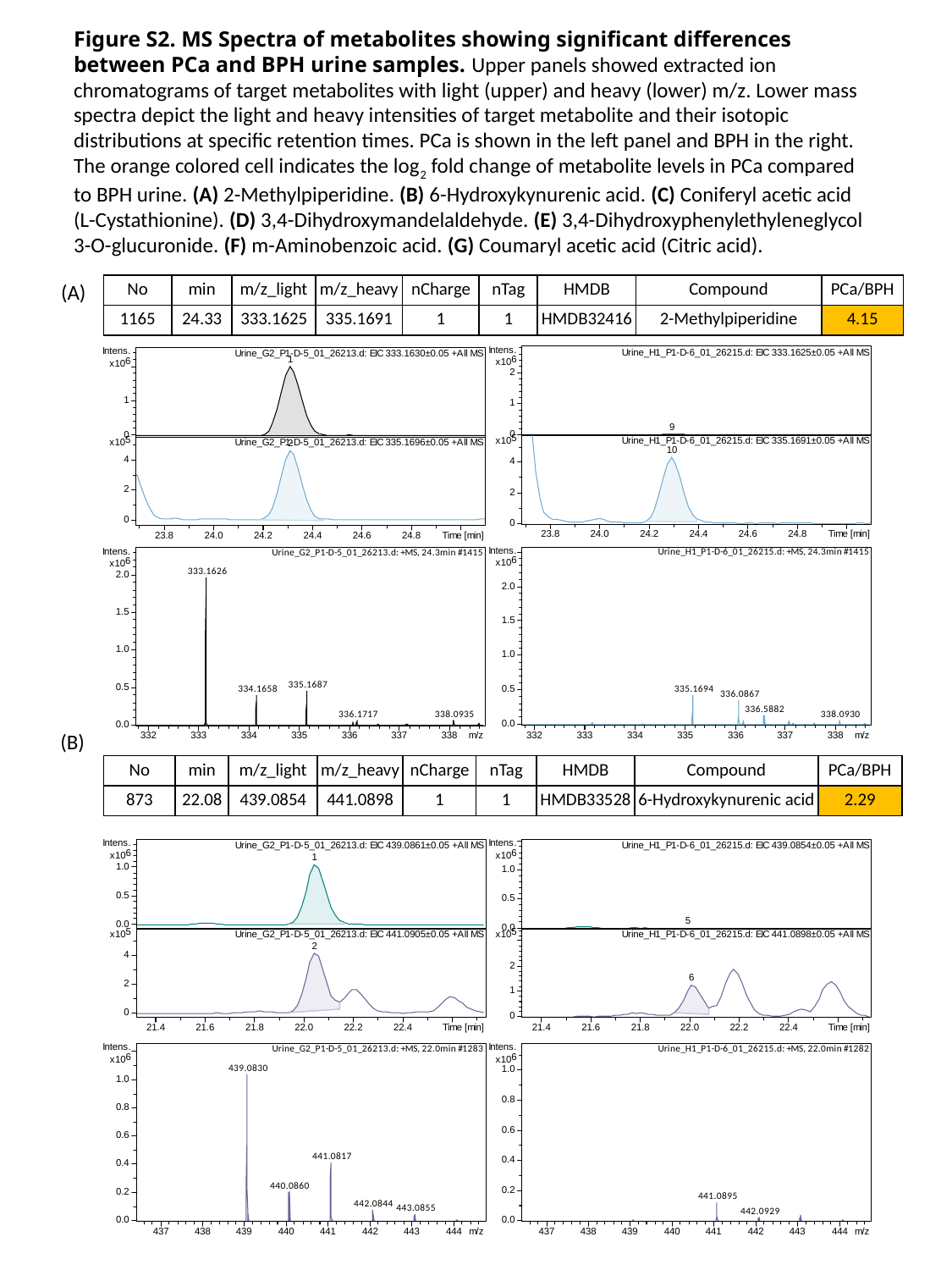

Figure S2. MS Spectra of metabolites showing significant differences between PCa and BPH urine samples. Upper panels showed extracted ion chromatograms of target metabolites with light (upper) and heavy (lower) m/z. Lower mass spectra depict the light and heavy intensities of target metabolite and their isotopic distributions at specific retention times. PCa is shown in the left panel and BPH in the right. The orange colored cell indicates the log2 fold change of metabolite levels in PCa compared to BPH urine. (A) 2-Methylpiperidine. (B) 6-Hydroxykynurenic acid. (C) Coniferyl acetic acid (L-Cystathionine). (D) 3,4-Dihydroxymandelaldehyde. (E) 3,4-Dihydroxyphenylethyleneglycol 3-O-glucuronide. (F) m-Aminobenzoic acid. (G) Coumaryl acetic acid (Citric acid).
(A)
| No | min | m/z\_light | m/z\_heavy | nCharge | nTag | HMDB | Compound | PCa/BPH |
| --- | --- | --- | --- | --- | --- | --- | --- | --- |
| 1165 | 24.33 | 333.1625 | 335.1691 | 1 | 1 | HMDB32416 | 2-Methylpiperidine | 4.15 |
(B)
| No | min | m/z\_light | m/z\_heavy | nCharge | nTag | HMDB | Compound | PCa/BPH |
| --- | --- | --- | --- | --- | --- | --- | --- | --- |
| 873 | 22.08 | 439.0854 | 441.0898 | 1 | 1 | HMDB33528 | 6-Hydroxykynurenic acid | 2.29 |

## Slide 3
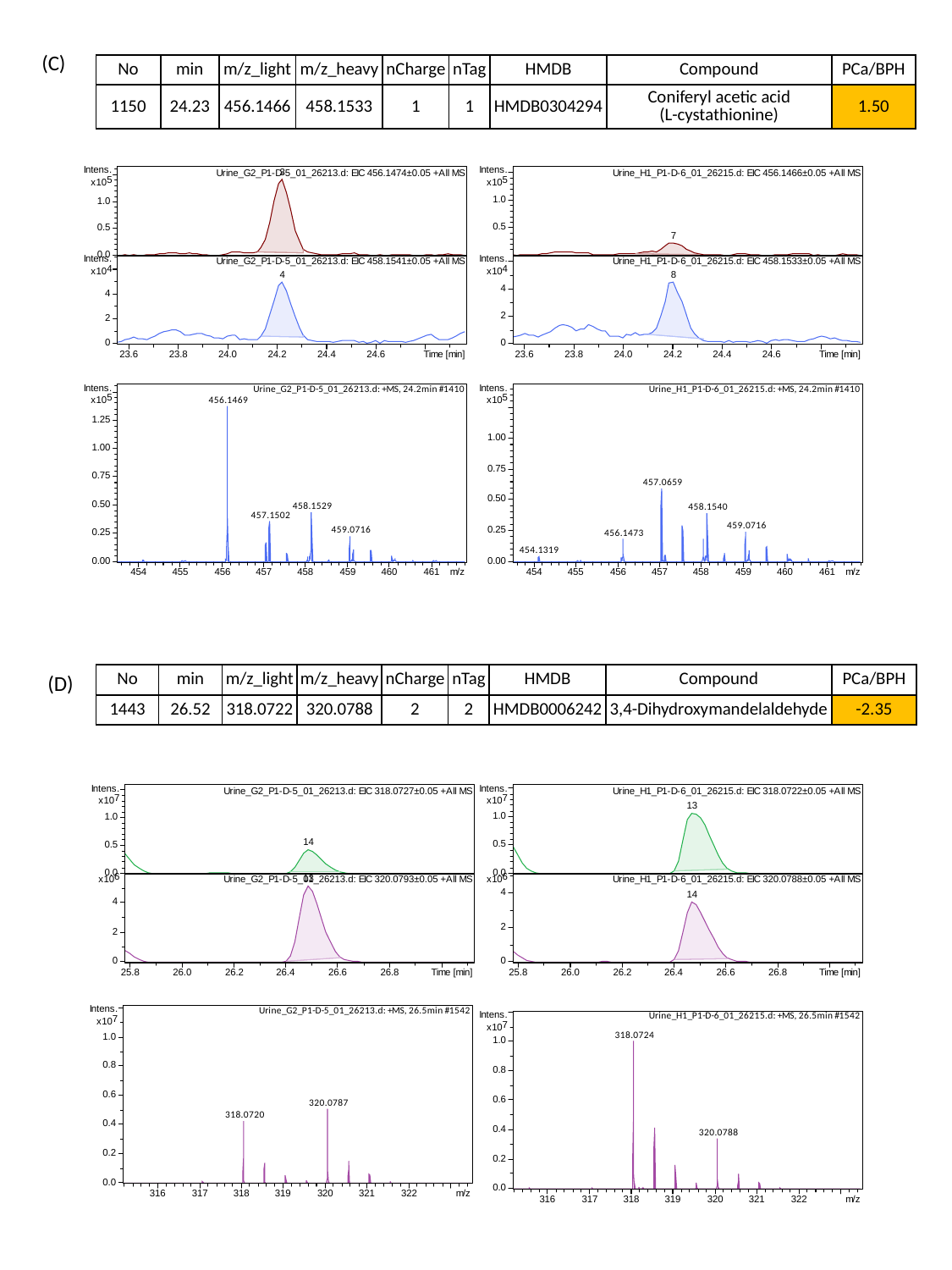

(C)
| No | min | m/z\_light | m/z\_heavy | nCharge | nTag | HMDB | Compound | PCa/BPH |
| --- | --- | --- | --- | --- | --- | --- | --- | --- |
| 1150 | 24.23 | 456.1466 | 458.1533 | 1 | 1 | HMDB0304294 | Coniferyl acetic acid (L-cystathionine) | 1.50 |
(D)
| No | min | m/z\_light | m/z\_heavy | nCharge | nTag | HMDB | Compound | PCa/BPH |
| --- | --- | --- | --- | --- | --- | --- | --- | --- |
| 1443 | 26.52 | 318.0722 | 320.0788 | 2 | 2 | HMDB0006242 | 3,4-Dihydroxymandelaldehyde | -2.35 |

## Slide 4
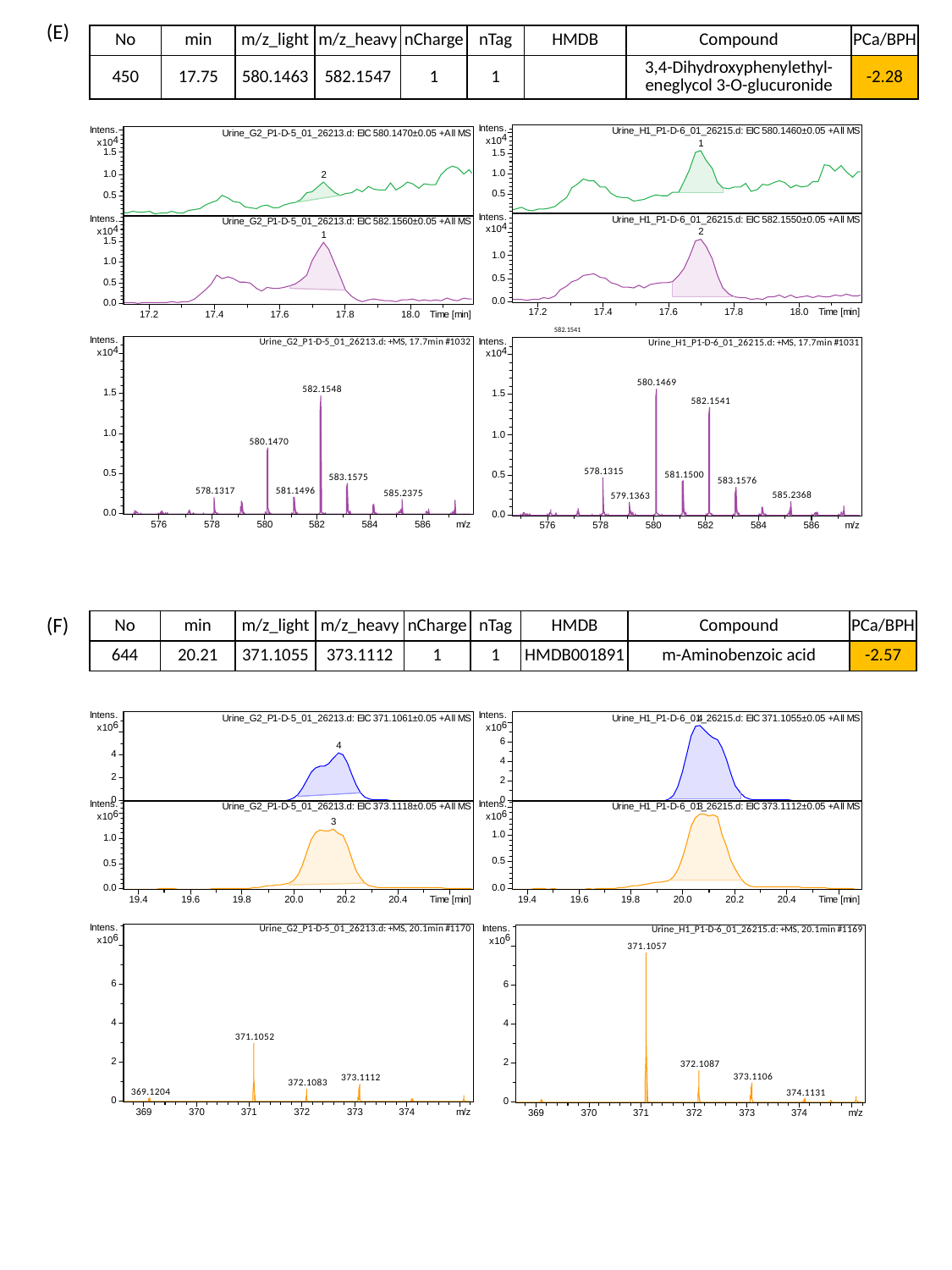

(E)
| No | min | m/z\_light | m/z\_heavy | nCharge | nTag | HMDB | Compound | PCa/BPH |
| --- | --- | --- | --- | --- | --- | --- | --- | --- |
| 450 | 17.75 | 580.1463 | 582.1547 | 1 | 1 | | 3,4-Dihydroxyphenylethyl-eneglycol 3-O-glucuronide | -2.28 |
582.1541
580.1386
(F)
| No | min | m/z\_light | m/z\_heavy | nCharge | nTag | HMDB | Compound | PCa/BPH |
| --- | --- | --- | --- | --- | --- | --- | --- | --- |
| 644 | 20.21 | 371.1055 | 373.1112 | 1 | 1 | HMDB001891 | m-Aminobenzoic acid | -2.57 |

## Slide 5
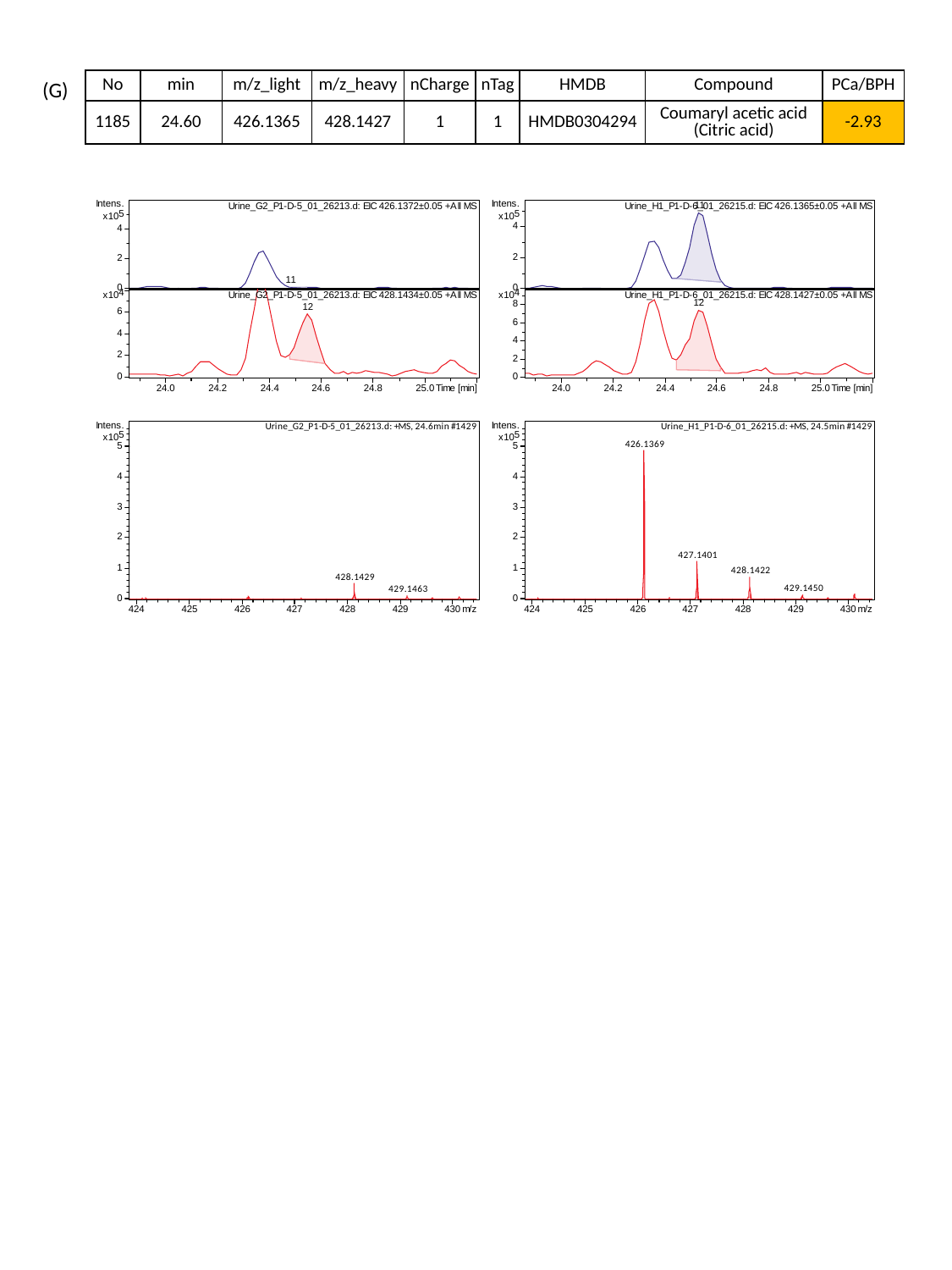

(G)
| No | min | m/z\_light | m/z\_heavy | nCharge | nTag | HMDB | Compound | PCa/BPH |
| --- | --- | --- | --- | --- | --- | --- | --- | --- |
| 1185 | 24.60 | 426.1365 | 428.1427 | 1 | 1 | HMDB0304294 | Coumaryl acetic acid (Citric acid) | -2.93 |
